# Enlarging Ventriculus Terminalis in a Patient With Polyarteritis Nodosa

**DOI:** 10.7759/cureus.14460

**Published:** 2021-04-13

**Authors:** Luke J Weisbrod, Collin Liu, Daniel Surdell

**Affiliations:** 1 Neurological Surgery, University of Nebraska Medical Center, Omaha, USA; 2 Neurosurgery, University of Nebraska Medical Center, Omaha, USA

**Keywords:** terminalis, terminal, ventriculus, ventricle, fifth, polyarteritis, nodosa, enlarging, conus, medullaris

## Abstract

Ventriculus terminalis (VT) is a cystic embryological remnant within the conus medullaris that normally regresses after birth. In rare cases, it may persist into adulthood and give rise to neurologic symptoms. The pathogenesis remains unclear but is thought to be related to failed embryonic regression with other proposed possible etiologies including vascular disturbances. We present an intriguing case of a slow-growing VT in a woman with progressive neurologic symptoms who experiences symptomatic relief following thoracic laminectomy and fenestration. Our case is the first to present a unique association with polyarteritis nodosa and only the third to report a case of documented enlargement of the VT over time successfully treated with surgical fenestration.

## Introduction

The ventriculus terminalis (VT) or “fifth ventricle” is a small ependymal-lined residual lumen in continuity with the central canal of the conus medullaris. VT is normally present in all humans during fetal development and typically regresses completely after birth [1-3]. Its persistence into adulthood represents a rare pathology [4]. In contrast to syringomyelia and intramedullary tumors, VT is found exclusively in the conus medullaris, is rounded in shape, and is non-contrast enhancing [3]. The pathogenesis of persistence of VT into adulthood remains unclear. Here, we present the case of a 57-year-old female with polyarteritis nodosa and an incidentally found VT that enlarges over time in relation to symptom onset.

## Case presentation

**Figure 1:**
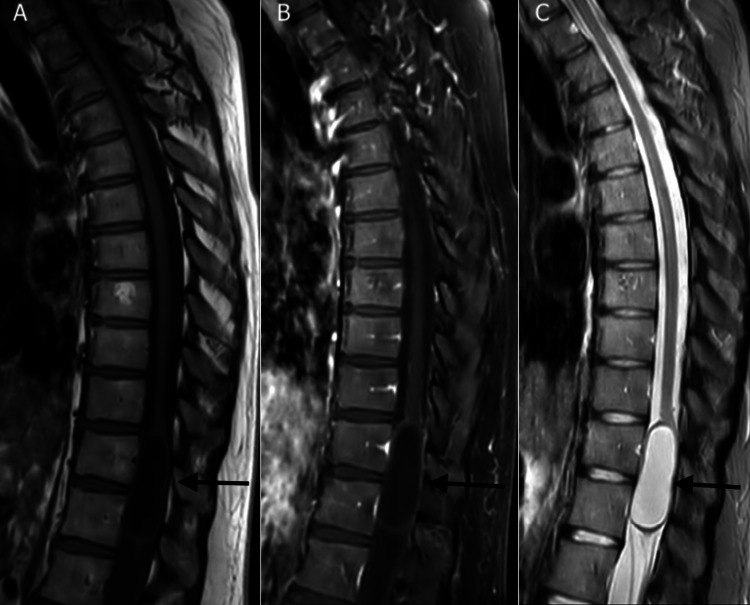
Sagittal MRI thoracic spine redemonstrating cystic lesion that has increased in size on follow-up imaging nine years later. (A) T1 non-contrast sequence; (B) T1 post-contrast; (C) T2.

**Figure 2:**
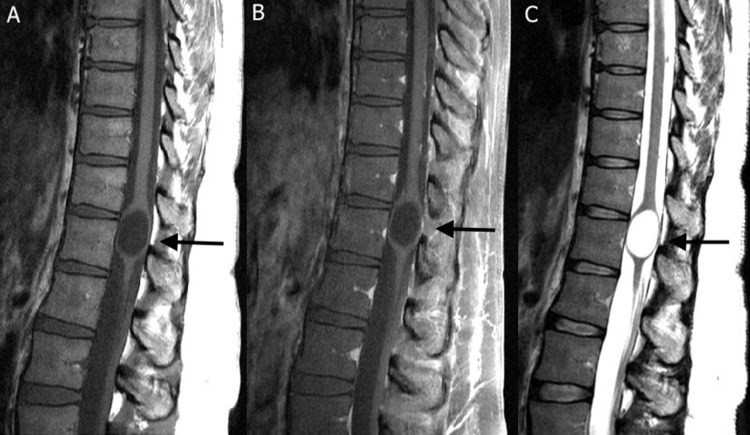
Sagittal MRI thoracic spine demonstrating a cystic lesion near the conus medullaris spanning thoracic level 11 to thoracic level 12. (A) T1 non-contrast sequence; (B) T1 post-contrast; (C) T2.

A 57-year-old female with a past medical history significant for polyarteritis nodosa on immunosuppression with Azathioprine presented to the neurosurgery clinic with progressive right lower extremity shooting pain, numbness and tingling over the past six months. On physical examination, she had trace weakness in right lower extremity dorsiflexion as well as right foot eversion with diminished vibratory and pin-prick sensation in her bilateral lower extremities. Magnetic resonance imaging (MRI) of the thoracic and lumbar spine with and without contrast was obtained which demonstrated a cystic lesion in the conus medullaris spanning the thoracic 11 to thoracic 12 levels (Figure 1). This cystic lesion was first identified nine years prior in 2011 (Figure 2). At the time of initial imaging in 2011, the patient did not have any neurologic complaints nor did she have any appreciable neurologic deficits on physical examination. A dedicated MRI of the thoracolumbar spine was obtained in 2011 due to an indeterminate finding observed in the conus medullaris on abdominal MRI for workup of abdominal pain. At the time of initial identification in 2011, due to the lack of neurologic symptoms and neurologic deficits, the cystic mass was determined to be an incidental finding with no further intervention pursued. On MRI thoracic spine with and without contrast at the time of her subsequent presentation in 2020, she was found to have interval enlargement (Figure 1) of the previously imaged conus medullaris cystic mass (Figure 2) which had enlarged over the course of the past nine years. Electromyography (EMG) and nerve conduction velocities (NCV) were obtained with normal results, making a peripheral neurologic process such as neuropathy or radiculopathy less likely. Based on the interval increase in the size of the conus medullaris lesion in the setting of normal EMG and NCV, it was felt the etiology of her current symptomatology and neurologic examination findings were related to the cystic conus medullaris lesion. She subsequently underwent a thoracic-11 to thoracic-12 laminectomy with cyst fenestration. She tolerated the surgery well and was discharged on postoperative day 3. Her pathology was negative for neoplasm and read as “minute fragments of glial tissue with no significant histopathological abnormality.” At the time of her four-week follow-up, her right lower extremity pain, numbness, tingling and strength had all significantly improved. Her four-month follow-up imaging remained pending at the time of submission of this article. 

## Discussion

The VT is an ependymal-lined cavity in continuity with the central canal of the conus medullaris. The VT is normally formed during fetal development between days 43 and 48 of embryogenesis and subsequently disappears during the regressive differentiation period [3]. While VT’s normally regress completely after birth, their persistence may occasionally be seen in children and has been incidentally detected in approximately 2.6% of children under five years of age [5]. In adults, it represents a very rare finding with only 68 cases reported in the literature managed with the surgical intervention [6].

The pathogenesis of persistence of VT into adulthood remains unclear. Proposed theories include dys-embryogenetic failed regression [7] with possibly etiologies related to vascular disturbances, ischemic necrosis of the spinal cord, compressive lesions, or diffuse inflammatory states [8]. Here, we have presented a possible association of VT with polyarteritis nodosa. PAN is a systemic necrotic vasculitis that affects small- and medium-sized arteries. Lesions of the nervous system are estimated to occur in 50% of cases of PAN [9], and in some cases, lesions of the peripheral or central nervous system may be the first presenting sign of disease [10-12]. Because descriptions of VT in adults have been limited to case reports and small case series, true associations are difficult to identify. To the best of our knowledge, this is the first case report of a patient with VT and PAN.

VT is typically identified on investigation for low back pain or neurological symptoms in the lower extremities, including urinary dysfunction [13]. In contrast to intra-medullary spinal cord tumors and syringomyelia, VT is rounded, not contrast-enhancing, and is exclusively found in the conus medullaris [3].

The optimal treatment for symptomatic VT, whether it be conservative or surgical remains uncertain. In an attempt to stratify patients into treatment paradigms, Ropert and Metral established a clinical classification system based on the limited literature (“cystic lesion of the VT classification” CLVT) [14]. This classification system was subsequently revised by Ganau et al, in which patients are categorized into CLVT type Ia (stable non-specific symptoms without a clear relation to VT), type Ib (non-specific but progressing symptoms), type II (focal neurologic deficit), and type III (sphincter disturbance) [4]. The CLVT system proposes that Ia lesions are best managed conservatively, while surgical intervention is recommended for the remaining categories [4]. Laminectomy and cyst fenestration is the most commonly described technique for treating persistent VT with a rate of symptomatic improvement ranging from 85% to 87% [15]. To the best of our knowledge, this is only the third case with documented enlargement of the VT over time [16,17] with patients experiencing symptomatic relief following surgical fenestration in all cases.

## Conclusions

We present an intriguing case of a slow-growing VT in a woman with progressive neurological symptoms. Despite its progressive growth, pathology was negative for neoplasm and she experienced symptomatic relief following thoracic laminectomy and fenestration. To the best of our knowledge, we are the first to report a possible association with polyarteritis nodosa and only the third to report a case of enlarging VT successfully treated with surgical fenestration.
